# MiR-125 inhibited cervical cancer progression by regulating VEGF and PI3K/AKT signaling pathway

**DOI:** 10.1186/s12957-020-01881-0

**Published:** 2020-05-30

**Authors:** Ke Fu, Ling Zhang, Rui Liu, Qi Shi, Xue Li, Min Wang

**Affiliations:** 1 Department of Gynecology and Obstetrics, The Fourth People’s Hospital of Liaocheng, City, Shandong Province, Liaocheng, China; 2 Department of Gynecology and Obstetrics, Liaocheng People’s Hospital, Liaocheng City, Shandong Province China; 3 Department of Postgraduate, Shandong First Medical University, Jinan City, Shandong Province China; 4 Department of Reproductive Genetics, Liaocheng People’s Hospital, No. 67, Dongchang West Road, Liaocheng City, 252000 Shandong Province China

**Keywords:** miR-125, VEGF, Cervical cancer, PI3K/AKT

## Abstract

**Background:**

MiR-125 has been shown to be involved in a variety of cancers, including cervical cancer (CC). Here, our goal was to explore miR-125 functional role and molecular mechanism in cervical cancer development and progression.

**Methods:**

qRT-PCR was employ to detect miR-125 and VEGF mRNA expression. Western blot was applied for testing protein levels (VEGF, E-cadherin, N-cadherin, vimentin, AKT, p-AKT, PI3K, and p-PI3K). MTT and transwell assays were used for detecting cervical cancer cell progression, including cell viability, migration, and invasion.

**Results:**

We observed that miR-125 was downregulated, whereas VEGF was upregulated in cervical cancer tissues and cell lines (CaSki and SiHa). MiR-125 inhibited the proliferation, invasion, and migration by targeting VEGF in cervical cancer. Moreover, miR-125 negatively regulated VEGF expression in cervical cancer tissues. Finally, we demonstrated that miR-520d-5p inhibited the activation of PI3K/AKT signaling pathway.

**Conclusion:**

In conclusion, the findings demonstrated that miR-125 inhibited cervical cancer progression and development by suppression VEGF and PI3K/AKT signaling pathway.

## Introduction

Cervical cancer (CC) is not only one of the most common tumors in the female genital tumors but also the most common cancer in women’s malignant tumors [1, 2]. According to the survey, the mortality of CC in China is the fourth in total cancer mortality, and the second in female cancer. The age of CC patients in China is 40-50 years old [3, 4]. Due to the recurrence and metastasis of the tumors, the treatment of CC patients is still poor [5]. Thus, to understand the internal mechanism associated with CC occurrence and the therapeutic method for CC is very necessary.

MicroRNAs (miRNA) are endogenous, non-coding small RNA which are approximately 22-25 nucleotides in length [6]. MiRNAs have been reported to modulate tumors development by degrading the translation of their target mRNAs. In CC progression, Dong P et al. displayed that miR-143 and miR-107 were involved in CC cell growth and invasion [7]. Besides, Shishodia G et al. revealed that miR-21 was higher in CC cells and associated with cervical carcinogenesis [8]. Moreover, Xie H et al. revealed that miR-194 promoted CC proliferation, invasion, and migration [9]. However, several miRNAs have been reported as tumor suppressor in CC progression, including miR-214, miR-9, and miR-129 [10–12]. MiR-125 was proved to be downregulated in CC and served as a biomarker for CC progression [13]. Based on these reports, we aimed to investigate the functional role of miR-125 in CC proliferation, invasion, migration, and tumor growth, which was rarely reported until now.

It is well known that miRNAs regulated their target mRNAs by binding to the 3′-UTR. Vascular endothelial growth factor (VEGF) is considered to be the most significant marker for hematologic malignancies screening [14]. VEGF acts as an oncogene in various solid tumors and determined as an irreplaceable tumor marker, including in colorectal cancer [15], glioblastoma [16], and breast cancer [17]. However, whether miR-125 regulated VEGF in CC progression was not reported until now. Here, we detected the VEGF role in CC progression and whether VEGF took part in CC progression modulated by miR-125.

PI3K/Akt signaling pathway is a key signaling pathway involved in multiple life activities and is involved in the regulation of cell division, differentiation, apoptosis, and other activities. PI3K/Akt pathway is associated significantly with tumor development and progression [18, 19]. Collectively, we investigated whether miR-125 modulated CC progression by VEGF through PI3K/Akt signaling pathway.

## Materials and methods

### CC tissue specimens

Fifty-eight paired fresh cervical cancer tissue specimens and matched adjacent normal tissue specimens were obtained from CC patients at the Fourth People’s Hospital of Liaocheng. No patients underwent chemotherapy, immunotherapy, hormonetherapy, or radiotherapy before sample collection. All the patients have signed the written informed consent. And the Institutional Review Board of Liaocheng People’s Hospital approved this study. The collected tissues were stored at −80 °C before further analysis. The clinical data were displayed in Table 1.

**Table 1:** Associations between miR-125 expression and clinicopathological characteristics

Characteristics	*n* = 58	miR-125		*P* value
		High (*n* = 28)	Low (*n* = 30)	
Age (years)				0.621
≥ 45	33	15	18	
< 45	25	13	12	
HPV 16/18 infection				0.301
Positive	41	18	23	
Negative	17	10	7	
Tumor size				0.031*
< 4 cm	25	8	17	
≥ 4 cm	33	20	13	
Histology				0.397
Squamous cell cancer	24	10	14	
Adenocarcinima and others	34	18	16	
Differentiation				0.061
Well and moderately	40	16	24	
Poor	18	12	6	
FIGO				0.018*
I	36	13	23	
II	22	15	7	
Lymph node metastasis				0.923
Negative	39	19	20	
Positive	19	9	10	

Statistical analyses were performed by the *χ*2 test

*P < 0.05 was considered significant

### Cell culture and cell transfection

Two cervical cancer cell lines (CaSki and SiHa) and the human cervical immortalized squamous cells (Ect1/E6E7) were obtained from ATCC. Dulbecco’s modified Eagle’s medium (DMEM; Hyclone, Logan, UT, USA) containing with 10% fetal bovine serum (FBS; Sigma-Aldrich, St. Louis, MO, USA), and antibiotic was applied for cell culture. The cells were maintained in a humidified incubator supplement with 5% CO_2_ at 37 °C.

MiR-125 mimic or inhibitor purchased from Shanghai GenePharma Co., Ltd. (Shanghai, China) was applied for over-expression or knockdown of miR-125. VEGF siRNA provided by Guangzhou RiboBio Co., Ltd. was used for silence VEGF. CaSki cells were selected for over-expression of miR-125; SiHa cells were selected for knockdown of miR-125. MiR-125 mimic, miR-125 inhibitor, or VEGF siRNA was transfected into CaSki and SiHa cells by using Lipofectamine 2000 reagent (Invitrogen) and the transfection was performed for 48 h.

### RT-PCR

Total RNAs were isolated from CC tissue specimens and cell lines (CaSki and SiHa) using TRIzol reagent (Invitrogen). Complementary DNA (cDNA) was synthesized using the PrimeScript RT reagent kit (TaKaRa, Dalian, China). MiScript reverse transcription kit (TaKaRa) was used for the reverse transcription from RNAs to cDNA. SYBR-Green PCR Master Mix (TaKaRa) was applied for conducting the reaction. The internal control was normalized by U6 and GAPDH. The gene mRNA expression was analyzed using 2^−ΔΔCt^ methods. The primers were shown in *Supplemental Table**1*.

### Western blot

Total proteins were exacted from CC tissues and cells with RIPA lysis buffer (Beyotime, Shanghai, China). The protein concentration was conducted using a BCA kit (Thermo Fisher Scientific, Inc.). Next, the proteins were separated by SDS-PAGE and transferred to the NC membranes. After blocking with 5% skimmed milk powder at 37 °C for 1 h, the membranes were incubated with primary antibodies at 4 °C overnight, followed by the secondary antibodies at 37 °C for 1 h. Finally, the proteins were detected by ECL kit (Pierce; Thermo Fisher Scientific, Inc.) and quantified by the Image J software (National Institutes of Health, Bethesda, MD, USA).

### Luciferase reporter assay

Firstly, the VEGF 3′-UTR-pGL3-reporter vector (Promega, Madison, WI, USA) was constructed. Then, CaSki cells were con-transfected with the vector and miR-125 mimic, SiHa cells were con-transfected with miR-125 inhibitor and vector. The luciferase activity was tested by the Dual-Luciferase Reporter Assay System (Promega) after transfection for 48 h.

### MTT assay

CC cells with miR-125 mimic, inhibitor, or VEGF siRNA were cultured in DMEM medium. Cells were seeded in a 96-well plate at a density of 3 × 103 cells/well and incubated for 0, 1, 2, 3, 4 days. MTT solution (5 mg/L, Sigma-Aldrich) was added and incubated for another 4 h. Then MTT solution was removed and the DMSO (Sigma-Aldrich) was added. Finally, we detected the optical density at a wavelength of 490 nm using a microplate reader.

### Transwell assay

The cells migration and invasion were detected by transwell assay as previously described [20]. Cells that migrated through the 8-μm sized pores and adhered to the lower surface of the filter were fixed with 4% paraformaldehyde, stained with 0.1% crystal violet, and counted under an inverted microscope (×200) to calculate their relative number.

### Xenograft tumor formation assays

The Animal Ethics Committee of the Fourth People’s Hospital of Liaocheng approved all animal testing procedures, the experiments were complied with the Helsinki Declaration and the Laboratory Animal Ethics Committee’s guidelines. CaSki cells treated with miR-125 mimic or NC were injected into the right flank of nude mice subcutaneously. Then, we recorded the tumor size and weight every 4 days for 28 days by a vernier caliper and electronic scale, respectively.

### Statistics analysis

The data were represented as mean ± SD from at least three times of experiments independently. Data was analyzed by the SPSS 22.0 statistical software, and the statistics was performed by GraphPad Prism 6 (version 6.0; GraphPad Software, USA). Student’s *t* test or one-way analysis of variance and Tukey’s post hoc test was applied for comparing the difference between two groups or more than two groups. *P* < 0.05 was considered as significant differences.

## Results

### MiR-125 was lowly expressed and VEGF was highly expressed in CC

**Fig. 1:**
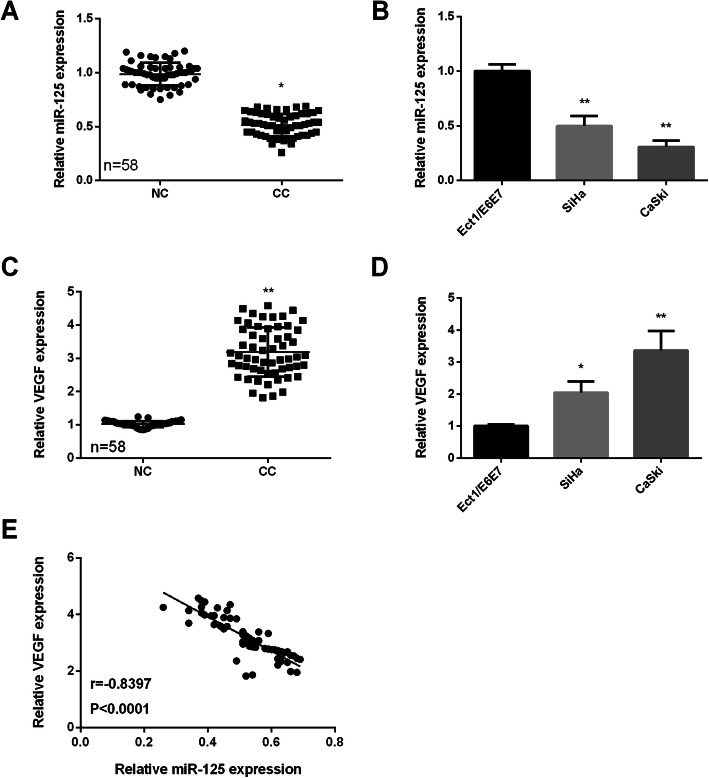
MiR-125 and VEGF expression in CC. **a** High expression of miR-125 in CC tissue specimens (*n* = 58). **b** High expression of miR-125 in CC cells. **c** Low expression of VEGF in CC tissue specimens (*n* = 58). **d** Low expression of VEGF in CC cells. **e** Negatively relationship between VEGF and miR-125. *P < 0.05, **P < 0.01

To know the role of miR-125 and VEGF in CC progression, their expression should be detected firstly in CC tissues and cells. As we saw in Fig.1a, miR-125 was lowly expressed in CC tissues compared to the normal tissues. Also, the expression of miR-125 was found lower in CC cell lines (CaSki and SiHa) compared to the normal Ect1/E6E7 cells (Fig. 1b). Through RT-qPCR analysis, we observed that, compared to adjacent normal tissues, VEGF expression was remarkably increased in CC tissues (Fig. 1a). Moreover, the VEGF expression in the human CC cell lines (CaSki and SiHa) was also significantly higher than that of Ect1/E6E7 cells. Based on these data, we investigated miR-125 and VEGF relationship. Results displayed that they were negatively related (*r* = −8397, *p* < 0.0001). These results demonstrated that dysregulation of miR-125 or VEGF might play different roles in CC progression.

### MiR-125 impeded CC viability, metastasis, and invasiveness

**Fig. 2:**
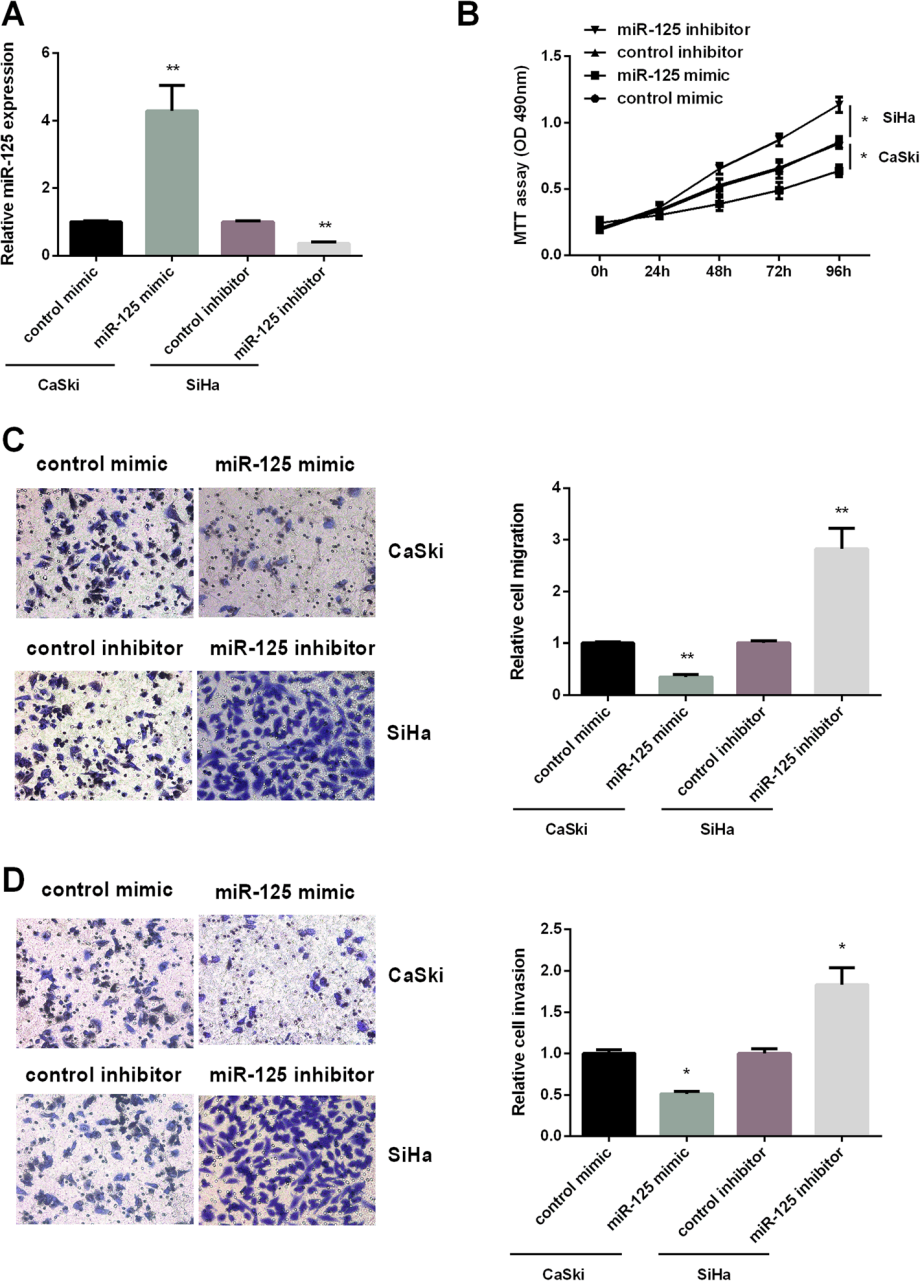
MiR-125 effect on CC progression. **a** High expression of miR-125 in CaSki cells and low expression of miR-125 in SiHa cells. **b** Hindrance effect of miR-125 mimic on CaSki cells viability and facilitating effect of miR-125 inhibitor on SiHa cells viability. **c** Hindrance effect of miR-125 mimic on CaSki cells migration and facilitating effect of miR-125 inhibitor on SiHa cells migration. Magnification ×200. **d** Hindrance effect of miR-125 mimic on CaSki cells invasion and facilitating effect of miR-125 inhibitor on SiHa cells invasion. Magnification ×200. *P < 0.05, **P < 0.01

To survey miR-125 effect on CC progression, miR-125 expression was increased in miR-125 mimic group than control mimic group; miR-125 expression was decreased in miR-125 inhibitor group than control inhibitor group. MiR-125 mimic was transfected into CaSki cells and miR-125 inhibitor was transfected into SiHa cells, due to miR-125 expression in CaSki cells was lower than in SiHa cells. As we expected in Fig. 2a, miR-125 expression was over-expressed in CaSki cells and low-expressed in SiHa cells. Moreover, we applied MTT and transwell assays to test miR-125 effect on CC cell progression. As we saw in Fig. 2b, the viability of CaSki cells was declined after treated with miR-125 mimic compared to that treated with control mimic, while SiHa cells viability was raised after treated with miR-125 inhibitor compared to that treated with control inhibitor. A transwell assay was applied to further evaluate the effect of miR-125 on cell migration and invasion. MiR-125 mimic decreased the number of migrated cells in CaSki cells compared to that treated with control mimic, miR-125 inhibitor increased the number of migrated cells in SiHa cells compared to that treated with control inhibitor (Fig. 2c). As shown in Fig. 2d, miR-125 mimic and miR-125 inhibitor have similar effects on CaSki and SiHa cell invasion. The findings above lead to a conclusion that miR-125 showed an impeding effect on CC cell progression.

### MiR-125 repressed tumor growth in vivo

**Fig. 3:**
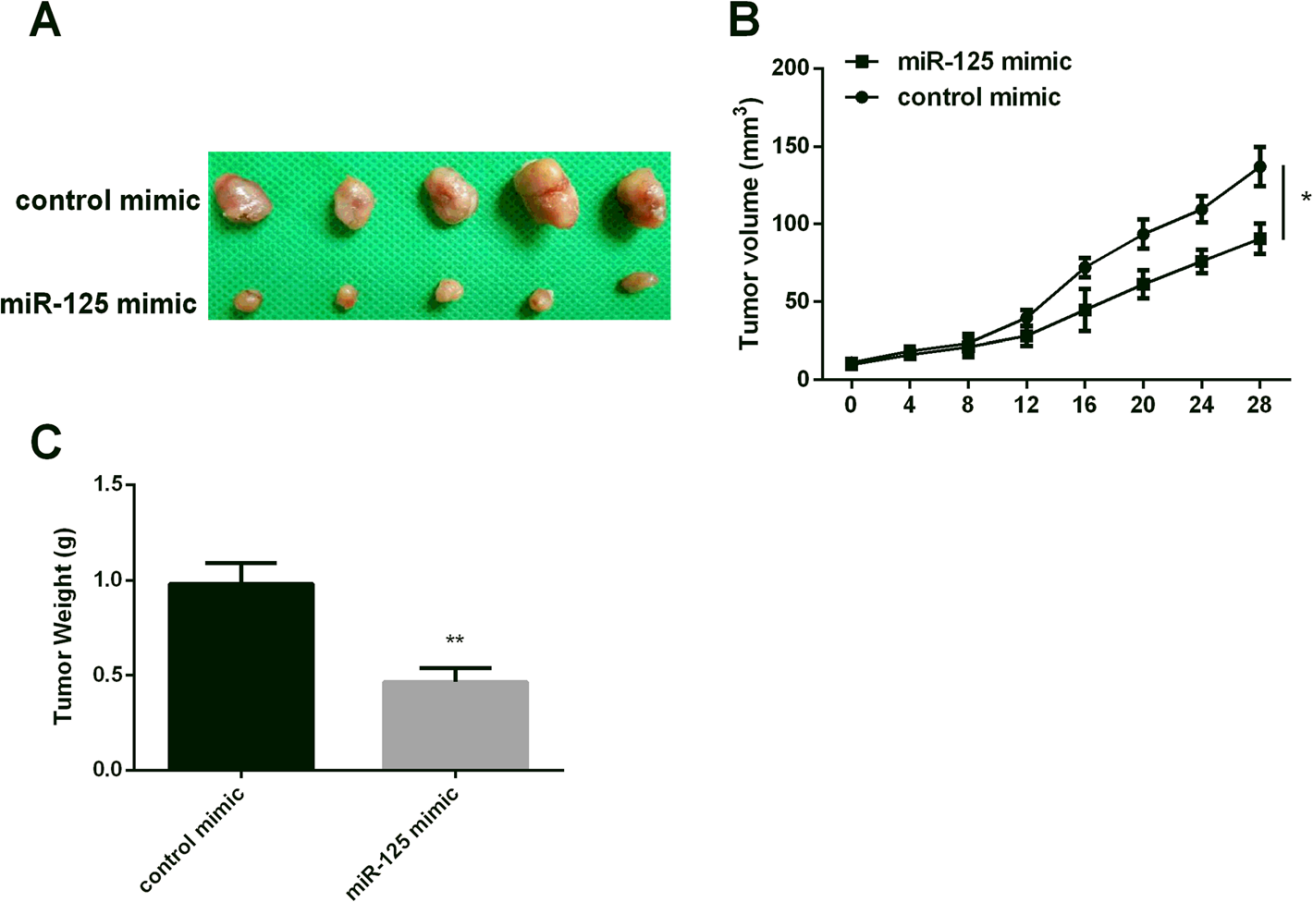
MiR-125 effect on tumor growth. **a** Tumor size of mice injected with miR-125 mimic cells. **b** Repressing effect of miR-125 mimic on tumor growth. **c** Repressing effect of miR-125 mimic on tumor weight. *P < 0.05, **P < 0.01

As miR-125 displayed a hindrance effect on CC progression in vitro. We then detected miR-125 effect on tumor growth in vivo. As Fig. 3a showed that miR-125 mimic blocked the growth of CC tumors compared to that of the control mimic group. Moreover, the tumors growth rate was more slowly than normal control (Fig. 3b). In addition, miR-125 upregulation repressed the increase in tumor weight compared to that of the control mimic group (Fig. 3c). These findings indicated that miR-125 hindered tumor growth.

### VEGF was the target of miR-125 in CC cells

**Fig. 4:**
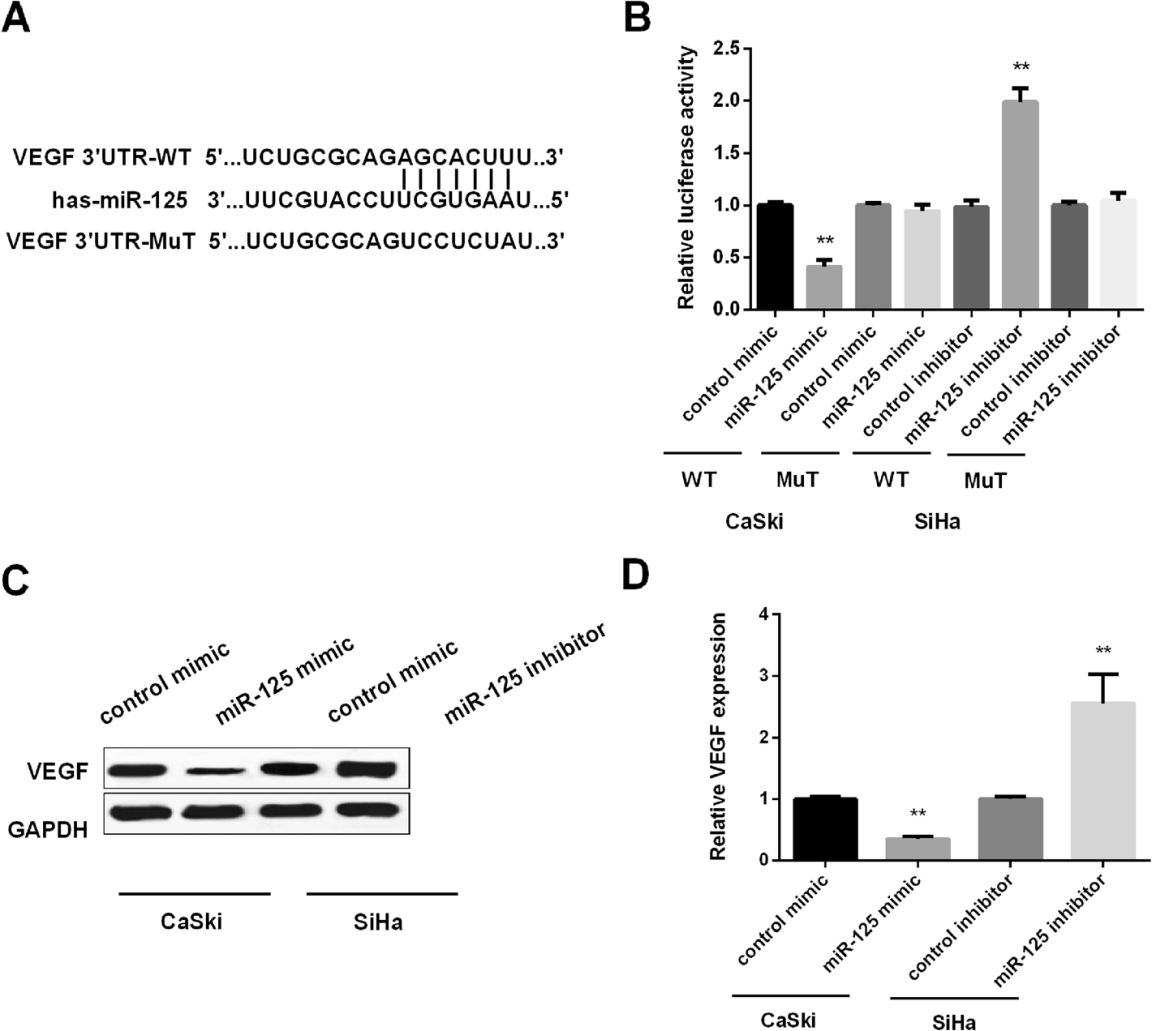
Confirmation the target gene of miR-125. **a** The binding sites of miR-125 and VEGF. **b** Impeding effect of miR-125 mimic on leciferase activity in CaSki cells and promoting effect of miR-125 inhibitor on SiHa cells. **c** The negatively regulated effect of miR-125 on VEGF protein level. **d** The negatively regulated effect of miR-125 on VEGF mRAN expression. **P < 0.01

As we found in Fig. 1, VEGF and miR-125 has the negative correlation in CC tissues and cells. We further surveyed their correlation in CC cells. As the TargetScan represented, VEGF contains a putative binding site for miR-125 (Fig. 4a). Moreover, luciferase reporter assay was applied for testing VEGF 3′-UTR luciferase activity in CaSki cells transfected with miR-125 mimic and SiHa cells with miR-125 inhibitor. We found that miR-125 mimic inhibited, while miR-125 inhibitor enhanced the luciferase activity (Fig. 4b). Furthermore, the high expression of miR-125 blocked the expression of VEGF, whereas the low expression of miR-125 facilitated VEGF expression in protein level (Fig. 4c). In mRNA level, miR-125 exhibited the same effect on VEGF expression (Fig. 4d). The above findings concluded that VEGF was the direct target of miR-125.

### VEGF overturned the effect of miR-125 on CC proliferation, invasion, and migration

**Fig. 5:**
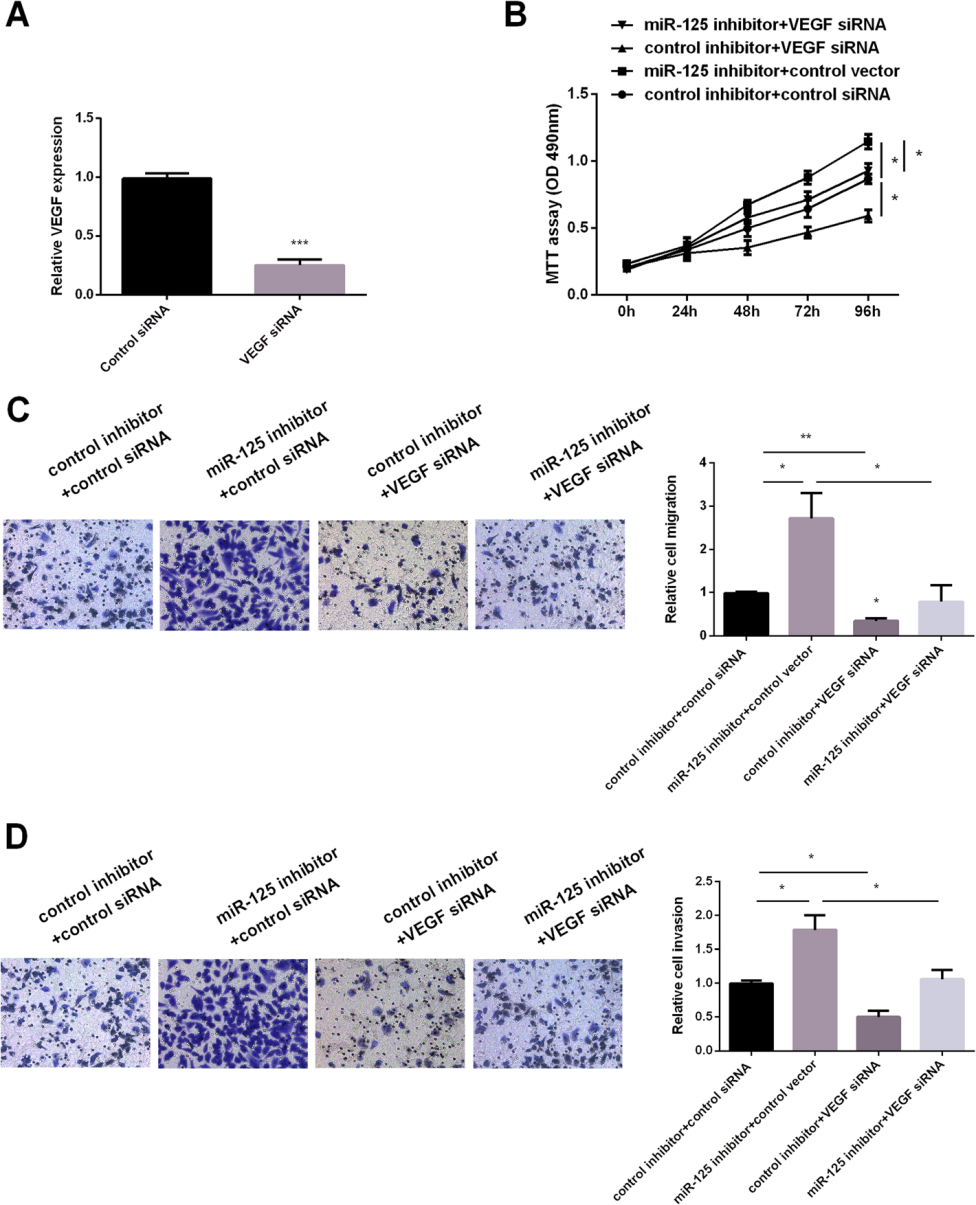
VEGF effect on CC progression regulated by miR-125. **a** Low expression of VEGF after knockdown of VEGF. **b** VEGF siRNA attenuated miR-125 inhibitor facilitating effect on CC cell viability. **c** VEGF siRNA attenuated miR-125 inhibitor facilitating effect on CC cell migration. Magnification ×200. **d** VEGF siRNA attenuated miR-125 inhibitor facilitating effect on CC cell invasion. Magnification ×200. *P < 0.05, **P < 0.01

Due to VEGF was over-expressed in CC tissues and cell lines, it was silenced by VEGF siRNA. As Fig. 5a displayed, the expression of VEGF was significantly decreased in VEGF siRNA group. Then, we applied MTT and transwell assays to test VEGF effect on CC cell progression. As we see in Fig. 5b, the viability of SiHa cells was inhibited after treated with VEGF siRNA and it was opposite to the effect of miR-125 inhibitor. Moreover, VEGF siRNA can reverse the promotion effect of miR-125 inhibitor on CC cell proliferation. For migration, VEGF siRNA displayed inhibitory effects and miR-125 inhibitor could reveal promotion effects. Moreover, VEGF siRNA could attenuate the effect of miR-125 inhibitor (Fig. 5c). As shown in Fig. 5d, the results showed that miR-125 downregulation significantly promoted the number of invasive cells, and VEGF siRNA showed the opposite effect. Additionally, VEGF siRNA could attenuate the effect of miR-125 inhibitor on invasion. The above findings lead to a conclusion that VEGF attenuated miR-125 inhibitor promotion effect on CC cell progression.

**Fig. 6:**
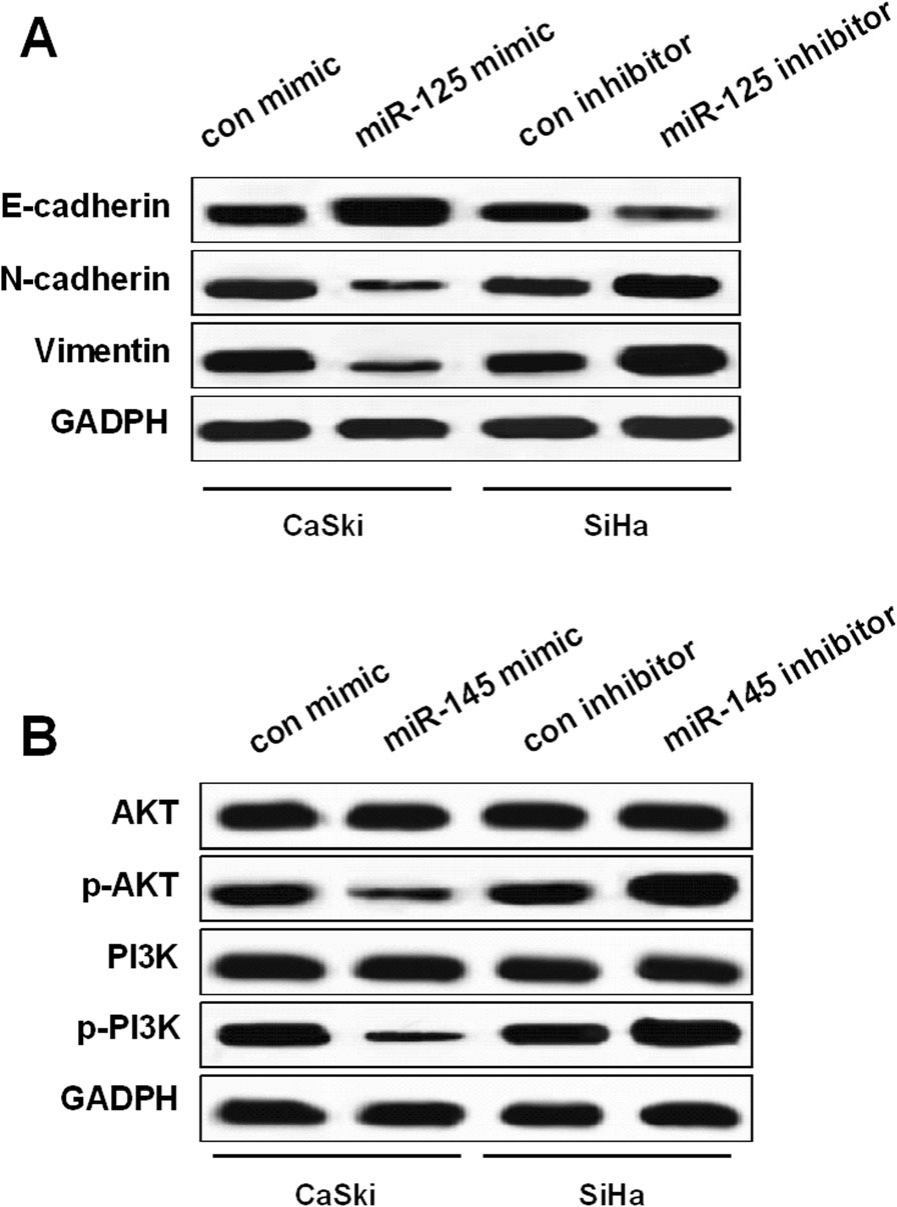
miR-125 effect on EMT and PI3K/AKT signaling pathway. **a** Suppression effect of miR-125 mimic on N-cadherin and vimentin levels in CaSki cells and promotion effect on E-cadherin level. However, promotion effect of miR-125 inhibitor on N-cadherin and vimentin levels in SiHa cells and inhibitory effect on E-cadherin level. **b** Suppression effect of miR-125 mimic on p-AKT and p-PI3K level in CaSki cells and promotion effect of miR-125 inhibitor on p-AKT and p-PI3K level in SiHa cells

### MiR-125 repressed EMT and PI3K/AKT signaling pathway

As we showed above, miR-125 impeded CC progression by targeting VEGF. Then, we explored whether PI3K/AKT signaling pathway was involved in CC progression modulated by miR-125. Results of Fig. 6 revealed that upregulation of miR-125 inhibited N-cadherin and vimentin levels in CaSki cells, while miR-125 downregulation enhanced N-cadherin and vimentin levels in SiHa cells. However, miR-125 showed the opposite effect on E-cadherin. Moreover, miR-125 upregulation inhibited p-AKT and p-PI3K expression in CaSki cells, whereas miR-125 downregulation enhanced their expression in SiHa cells. These data demonstrated that miR-125 suppressed EMT and PI3K/AKT signaling pathway in CC cells.

## Discussion

It is reported that the expression of many miRNAs is imbalanced in CC, the abnormal regulation of miRNAs is related to the occurrence and development of CC [21]. It is worth noting that exploring CC potential mechanism development may contribute to early diagnosis and effective treatment [22]. Here, in the study, we survey miR-125 role and its underlying mechanism in CC progression. The findings demonstrated that miR-125 served as a tumor suppressor in CC.

MiR-125 dysregulation was associated with tumor development and progression. For instance, it was highly expressed in lung cancer and implicated in NSCLC patient survival [23]. Also, miR-125 was upregulated in esophageal adenocarcinoma and associated with poor prognosis [24]. However, miR-125 was under-expressed in glioma and regulated cell growth and invasion [25]. Besides, Chen H showed that miR-125 downregulation acted as a potential biomarker for colorectal cancer treatment [26]. In this study, we revealed that miR-125 was low-expressed in CC and repressed cell proliferation, invasion, and migration.

To understand miR-125 role in CC progression, it is very important for confirming its direct target, which may help identify the promising treatments. Therefore, we firstly used TargetScan to predict the possible targets of miR-125. Among the candidate target genes, VEGF was selected for further analysis. VEGF is well known to function as an oncogene in varieties of tumors [27, 28]. Also, it acted as the target of some miRNAs in modulating tumor development and progression. For example, it served as the target of miR-29c in suppressing lung progression [29]. Moreover, miR-146a displayed suppression effect on hepatocellular carcinoma metastasis via targeting VEGF [30]. In addition, VEGF was also reported as a target of miR-125 in regulating colorectal cancer cell growth [31]. Here, we applied the luciferase reporter assay to confirm VEGF as the direct target of miR-125 in CC cells. Furthermore, miR-125 negatively regulated VEGF expression. In addition, we also detected that VEGF siRNA revealed an impeding effect on CC progression, which was opposite to the miR-125 inhibitor effect.

PI3K/AKT signaling pathway is one of the best-characterized kinase cascades in cancer cell biology and plays a central role in the carcinogenesis and maintenance of cancer [32, 33]. In cervical cancer, PI3K/AKT signal is critical in cell differentiation, proliferation, survival, migration, and apoptosis [34, 35]. Previous studies have shown that PI3K/Akt signaling pathway is closely associated with the occurrence and development of CC [36], and the pathway has become a potential target for the prevention and treatment of CC [37, 38]. As hypothesized, miR-125 overexpression decreased the expression of p-PI3K and p-AKT, while miR-125 knockdown showed opposite effects. Based on these results, we inferred that miR-125 can inhibit PI3K/AKT signaling pathway in CC cells.

In conclusion, miR-125 was under-expressed in CC and miR-125 upregulation repressed CC progression. However, VEGF was highly expressed in CC and it displayed the opposite effect to miR-125. Moreover, VEGF was confirmed as the target of miR-125 and miR-125 blocked the activation of PI3K/AKT pathway in CC cells. Correctively, miR-125 repressed CC progression by targeting VEGF through PI3K/AKT pathway.

## Supplementary information

**Supplemental Table 1.** Primer sequences for RT-PCR.

## Data Availability

All data generated or analyzed during this study are included in this published article.
