# Supplementary material for: MiR-125 inhibited cervical cancer progression by regulating VEGF and PI3K/AKT signaling pathway
Source: World J Surg Oncol. 2020 May 30;18:115. doi: 10.1186/s12957-020-01881-0 (PMC7261381; doi:10.1186/s12957-020-01881-0)
Supplement: Supplementary file 1 — Supplemental Table 1. Primer sequences for RT-PCR. [file 12957_2020_1881_MOESM1_ESM.docx]

**Supplemental Table 1. Primer sequences for RT-PCR**

| Gene | Primer sequences |
| --- | --- |
| GAPDH | Forward primer: 5′-GCACCGTCAAGGCTGAGAAC-3′  Reverse primer: 5′-ATGGTGGTGAAGACGCCAGT-3′ |
| U6 | Forward primer: 5′- CTCGCTTCGGCAGCACATATACT-3′  Reverse primer: 5′- ACGCTTCACGAATTTGCGTGTC-3′ |
| miR-125 | Forward primer: 5′- GCUCCCUGAGACCCUAAC-3′  Reverse primer: 5′- CAGTGCAGGGTCCGAGGT-3′ |
| VEGF | Forward primer: 5′- GCCTTAGGACACCATACCGATG-3′  Reverse primer: 5′- GCTGCCCCAGGGAACAAAGTTG-3′ |
